# Supplementary material for: IGLoo: Profiling the Immunoglobulin Heavy chain locus in Lymphoblastoid Cell Lines with PacBio High-Fidelity Sequencing reads
Source: bioRxiv. 2024 Jul 23:2024.07.20.604421. Preprint. [Version 1] doi: 10.1101/2024.07.20.604421 (PMC11291057; doi:10.1101/2024.07.20.604421)
Supplement: 1 [file NIHPP2024.07.20.604421V1-supplement-1.pdf]

bioRxiv preprint doi: https://doi.org/10.1101/2024.07.20.604421; this version posted July 23, 2024. The copyright holder for this preprint (which was not certified by peer review) is the author/funder, who has granted bioRxiv a license to display the preprint in perpetuity. It is made available under aCC-BY 4.0 International license.

Supplementary Material

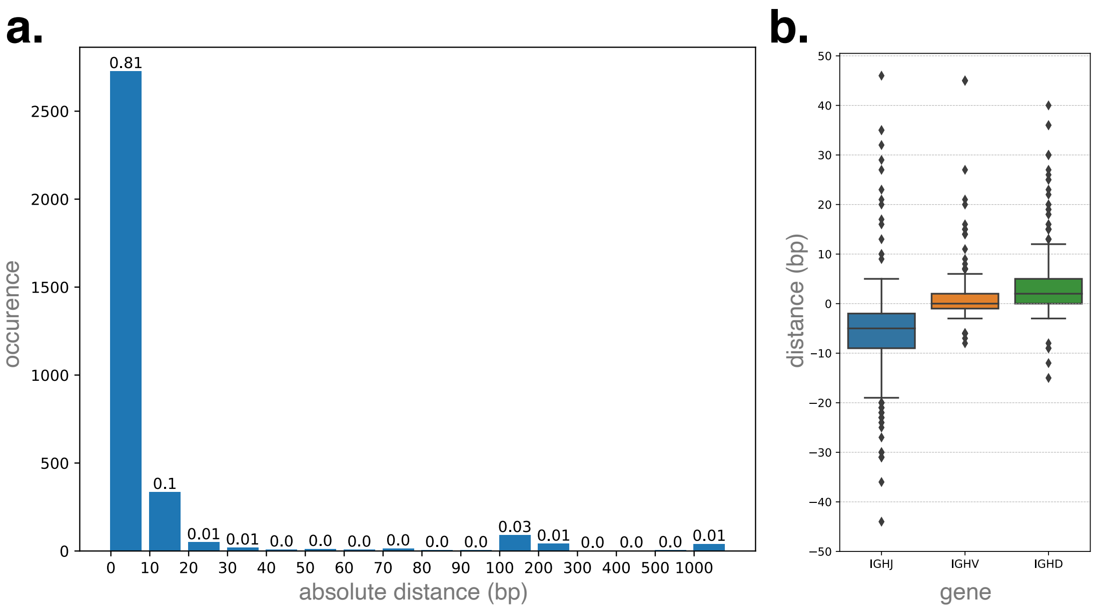

Figure S1: The distribution of the distance of the $3,357$ split site to RSSs. **a.** the histogram of the distribution of the absolute distance (bp) to the closest RSS, the first bin shows the occurrence of distance between 0 to 10, while the last bin shows the occurrence of the distance over 1000. **b.** the distribution of the distance (bp) to RSS stratified by IGHJ, IGHV, and IGHD genes. We only show the cases with distance < 50 bp. The distance of J genes are counted on its $5'$ end, while the distance of V and D genes are counted from their $3'$ end.

bioRxiv preprint doi: https://doi.org/10.1101/2024.07.20.604421; this version posted July 23, 2024. The copyright holder for this preprint (which was not certified by peer review) is the author/funder, who has granted bioRxiv a license to display the preprint in perpetuity. It is made available under aCC-BY 4.0 International license.

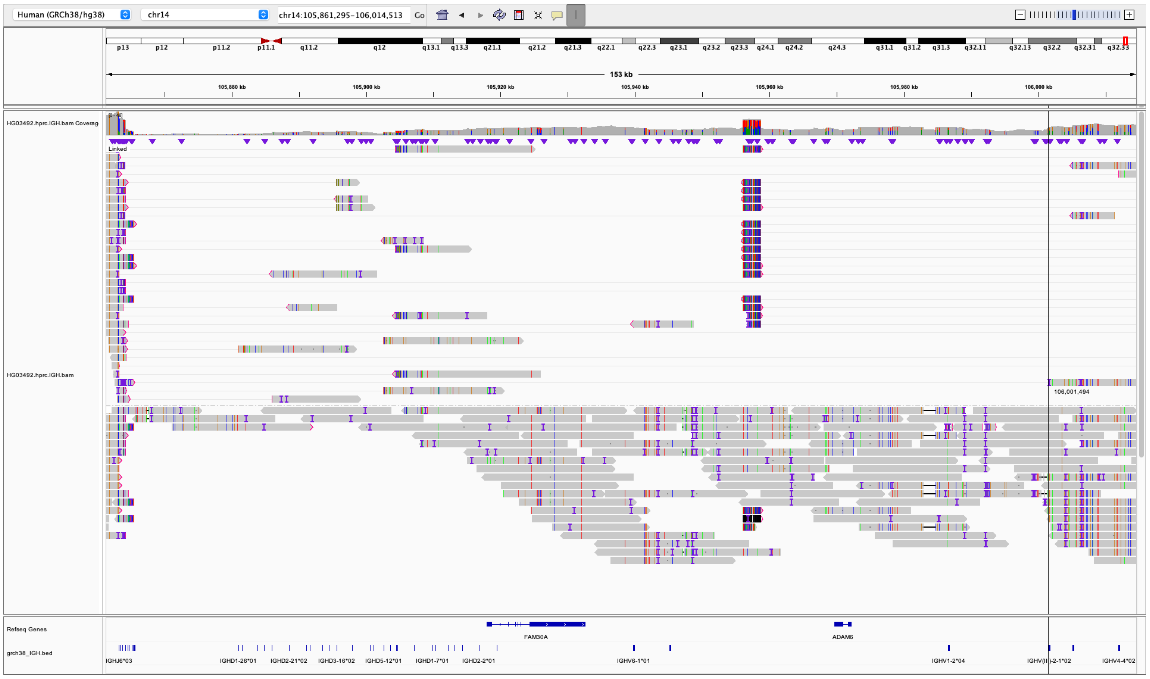

Figure S2: The complete V(D)J recombination event using the pseudogene *IGHV(III)-2-1*.

bioRxiv preprint doi: https://doi.org/10.1101/2024.07.20.604421; this version posted July 23, 2024. The copyright holder for this preprint (which was not certified by peer review) is the author/funder, who has granted bioRxiv a license to display the preprint in perpetuity. It is made available under aCC-BY 4.0 International license.

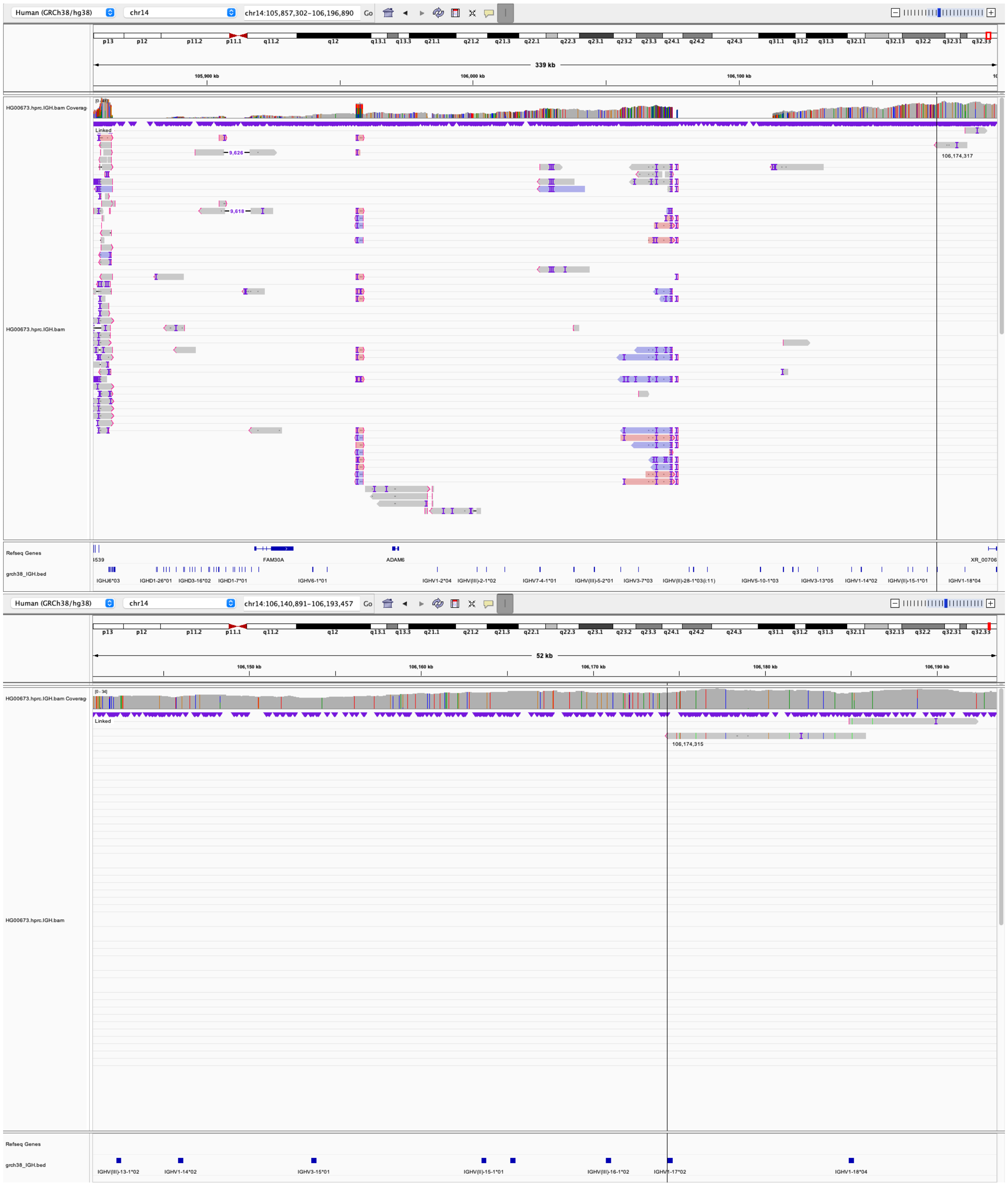

Figure S3: The bird eye view (**top**) and zoom in view (**bottom**) of the complete V(D)J recombination event using the pseudogene *IGHV1-17*.

bioRxiv preprint doi: https://doi.org/10.1101/2024.07.20.604421; this version posted July 23, 2024. The copyright holder for this preprint (which was not certified by peer review) is the author/funder, who has granted bioRxiv a license to display the preprint in perpetuity. It is made available under aCC-BY 4.0 International license.

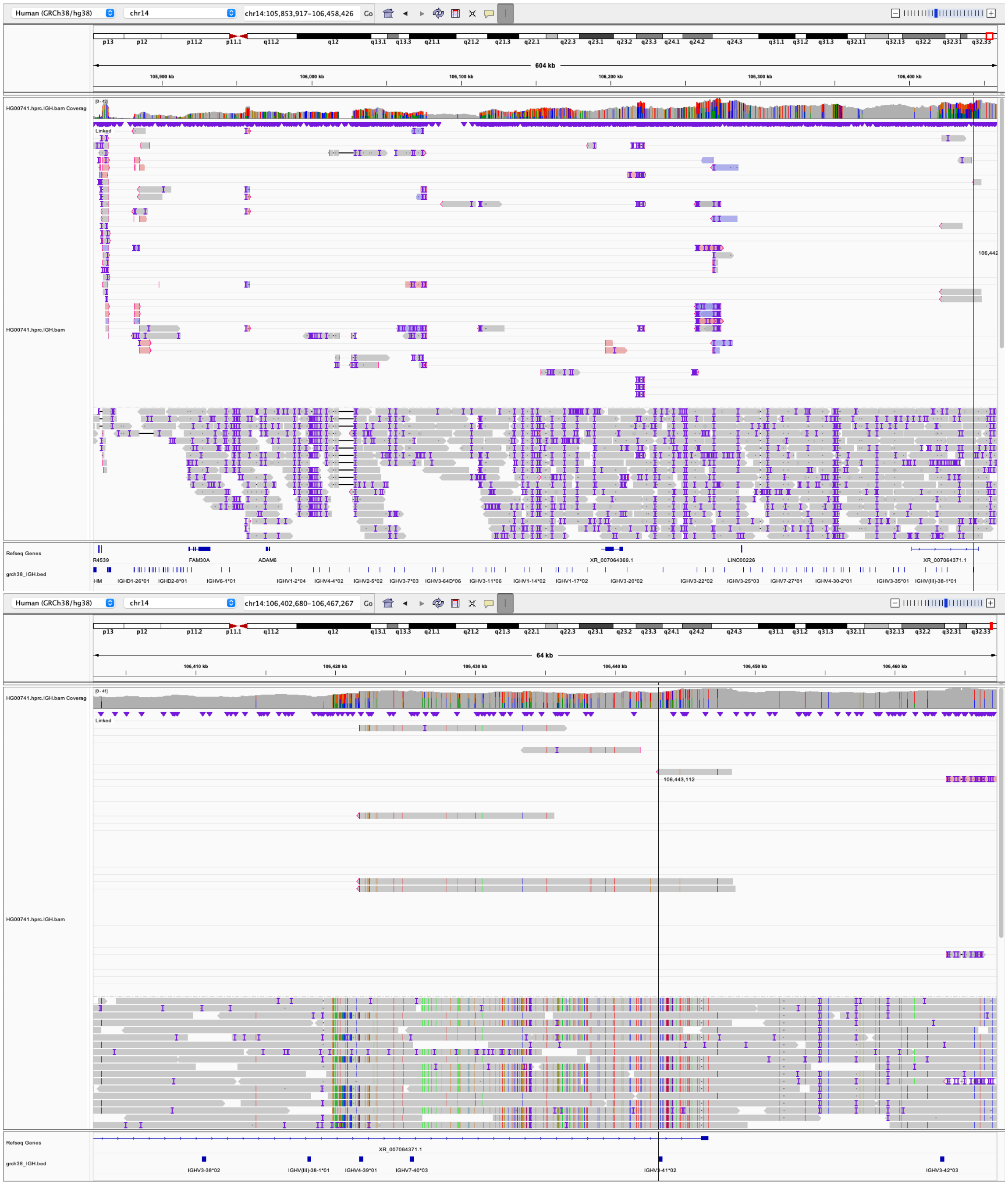

Figure S4: The bird eye view (**top**) and zoom in view (**bottom**) of the complete V(D)J recombination event using the pseudogene *IGHV3-41*.

bioRxiv preprint doi: https://doi.org/10.1101/2024.07.20.604421; this version posted July 23, 2024. The copyright holder for this preprint (which was not certified by peer review) is the author/funder, who has granted bioRxiv a license to display the preprint in perpetuity. It is made available under aCC-BY 4.0 International license.

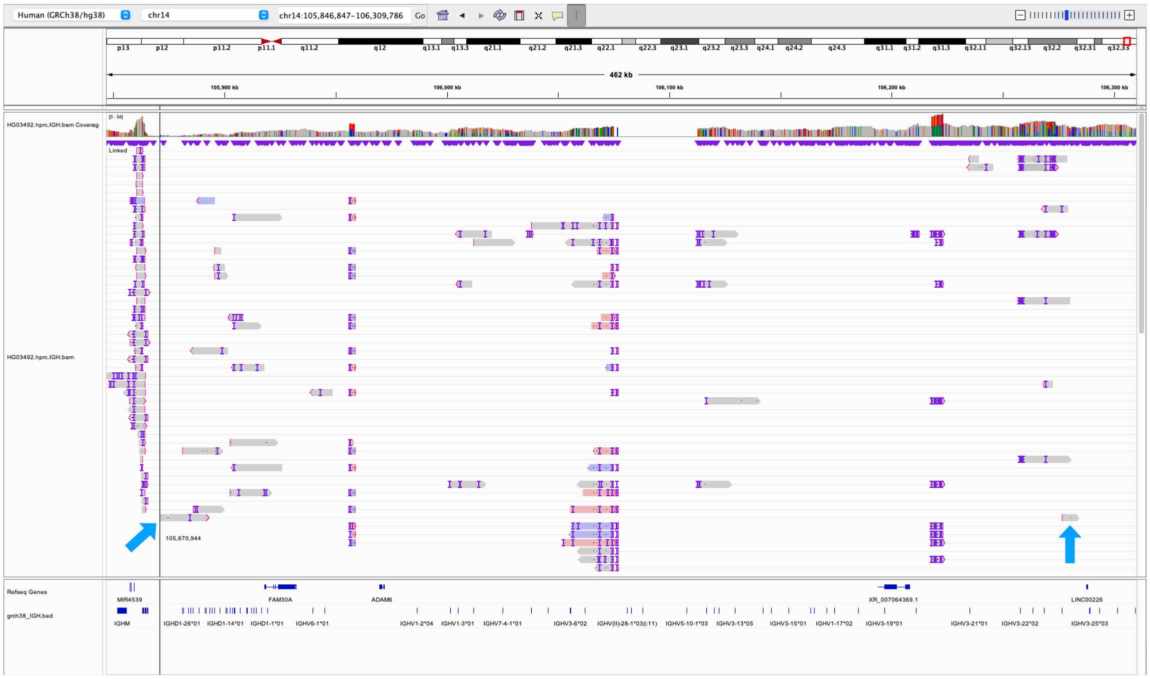

Figure S5: The only V-D only partial recombination event we observed in HPRC from individual HG03492. The blue arrows indicate the reads with a long deletion across the gene *IGHD6-19* and *IGHV1-24*. Note that the 3′ end segments of the read stretch to the position between IGHD and IGHJ loci.

bioRxiv preprint doi: https://doi.org/10.1101/2024.07.20.604421; this version posted July 23, 2024. The copyright holder for this preprint (which was not certified by peer review) is the author/funder, who has granted bioRxiv a license to display the preprint in perpetuity. It is made available under aCC-BY 4.0 International license.

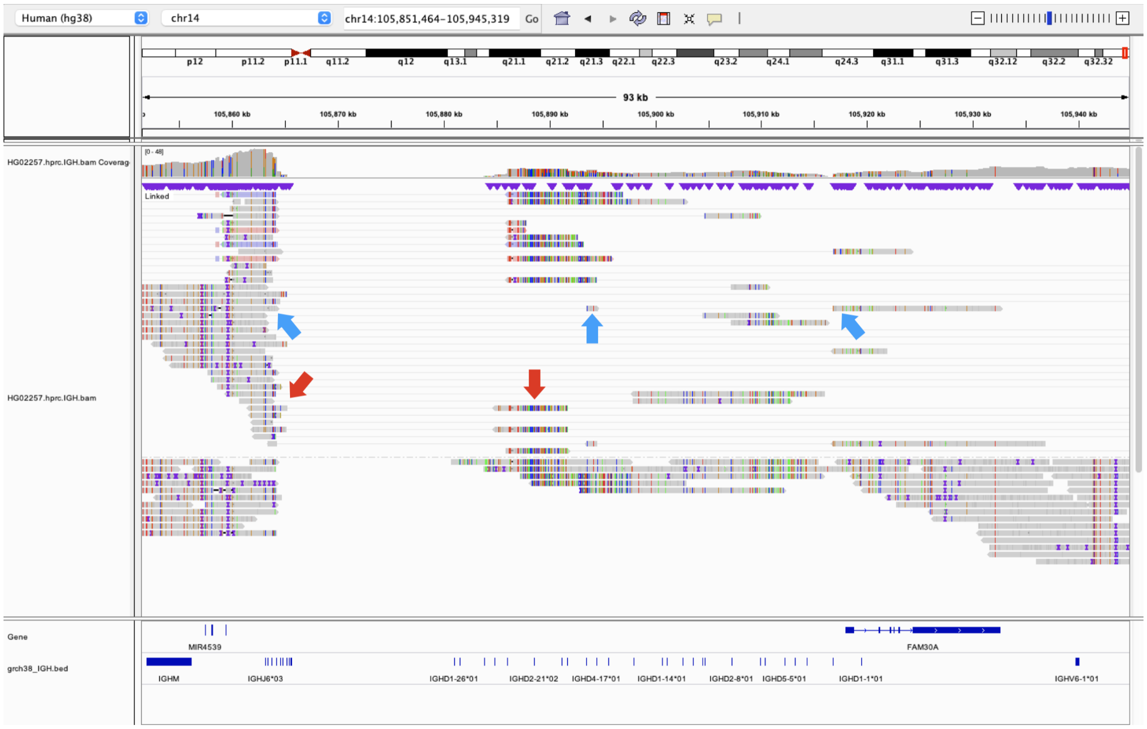

Figure S6: Two non-canonical recombination events of the individual HG02557. The blue arrows indicate the read with two long deletions crossing (*IGHJ4*, *IGHD5-18*) and (*IGHD4-17*, *IGHD2-2*). The red arrows show another read with two long deletions crossing (*IGHJ2*, *IGHD4-23*) and (*IGHD6-19*, *IGHV3-49*). The *IGHV3-49* end is beyond the scope of the screenshot.

bioRxiv preprint doi: https://doi.org/10.1101/2024.07.20.604421; this version posted July 23, 2024. The copyright holder for this preprint (which was not certified by peer review) is the author/funder, who has granted bioRxiv a license to display the preprint in perpetuity. It is made available under aCC-BY 4.0 International license.

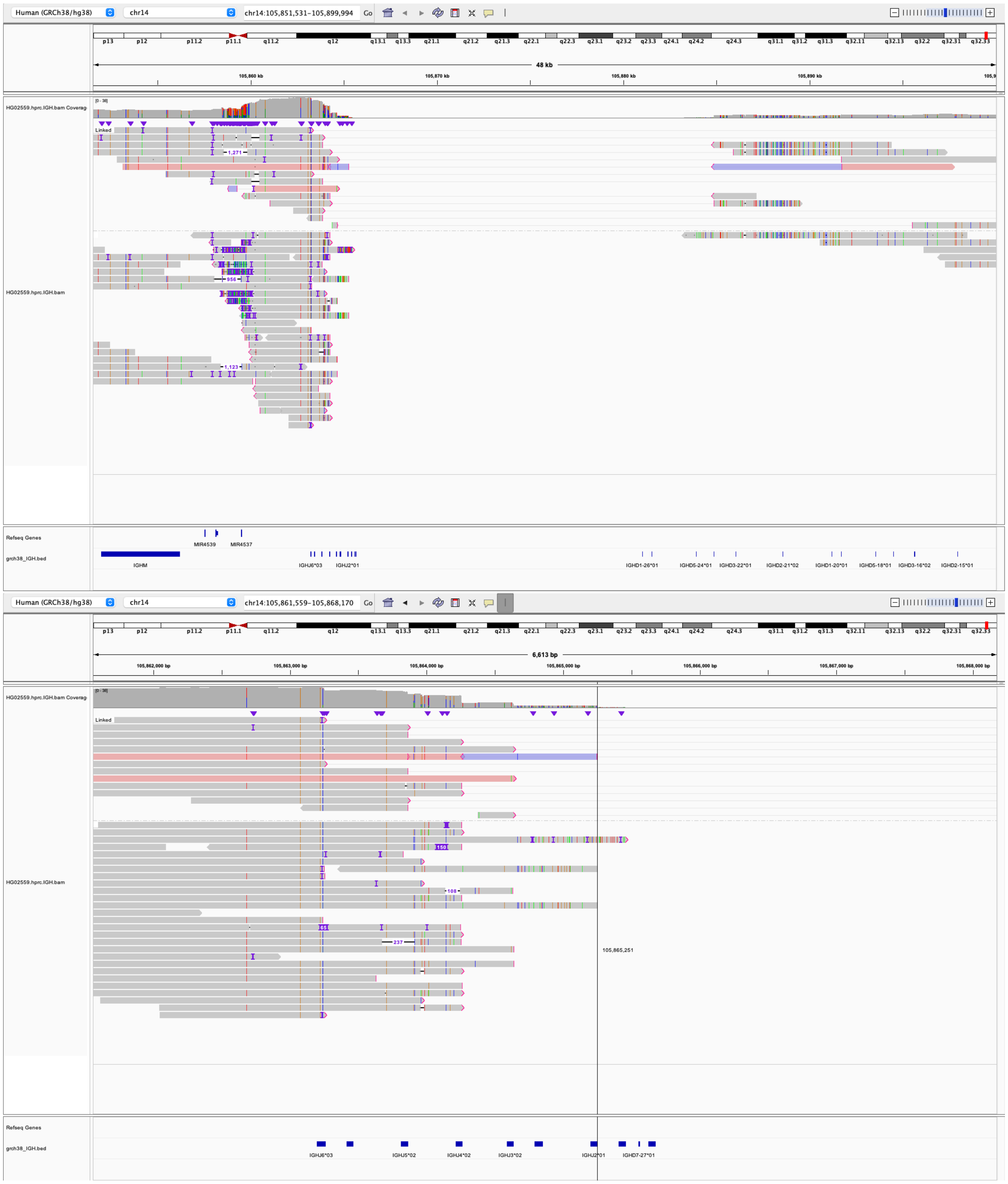

Figure S7: The bird eye view and the zoom in view of the individual HG02559 with an inverted J sequence event. The non-canonical recombination event involved an inversion between *IGHJ2* and *IGHJ4*, the sequence in between the two genes are inverted as the red and blue sequence shown.

bioRxiv preprint doi: https://doi.org/10.1101/2024.07.20.604421; this version posted July 23, 2024. The copyright holder for this preprint (which was not certified by peer review) is the author/funder, who has granted bioRxiv a license to display the preprint in perpetuity. It is made available under aCC-BY 4.0 International license.

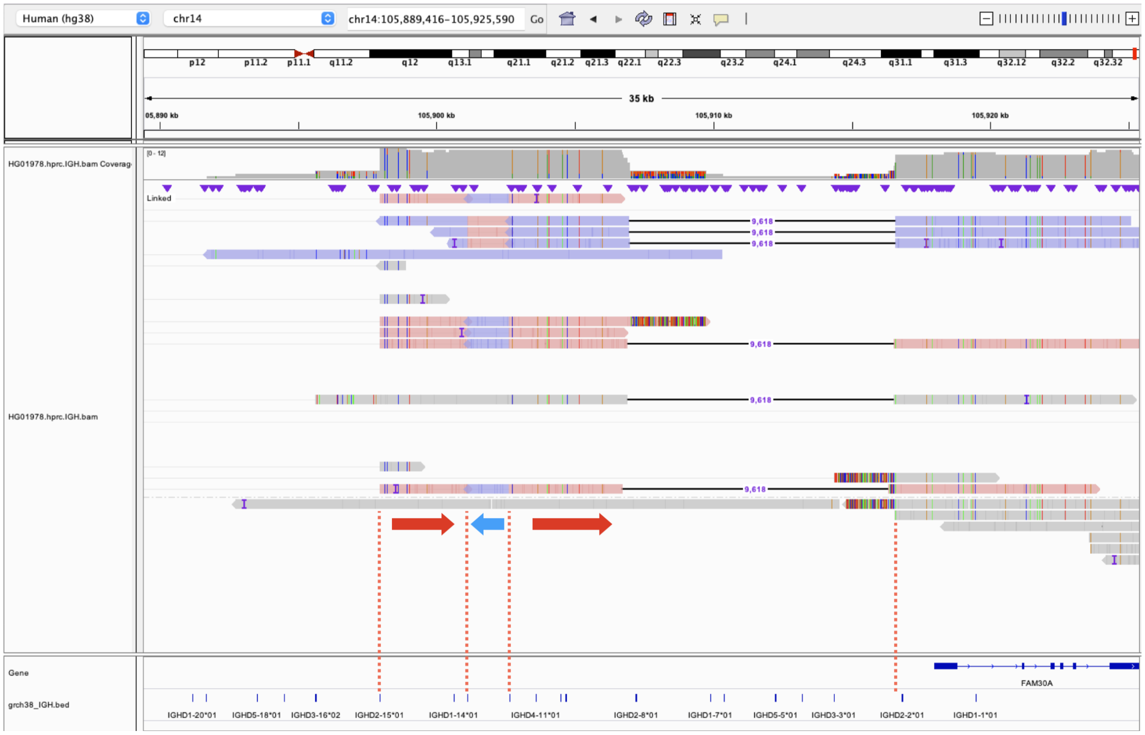

Figure S8: The non-canonical recombination event that involved an inversion between *IGHD6-13* and *IGHD5-12*. The sequence in between the two genes, which indicated by the blue arrow, is inverted. In the raw read, the head of the left side red arrow connects to the tail of the blue arrow, and the blue arrowhead connects to the tail of the right side red arrow. The *IGHD6-13* gene is on the arrowtail of the blue arrow, which is right next to the *IGHD5-12* gene on raw read.

bioRxiv preprint doi: https://doi.org/10.1101/2024.07.20.604421; this version posted July 23, 2024. The copyright holder for this preprint (which was not certified by peer review) is the author/funder, who has granted bioRxiv a license to display the preprint in perpetuity. It is made available under aCC-BY 4.0 International license.

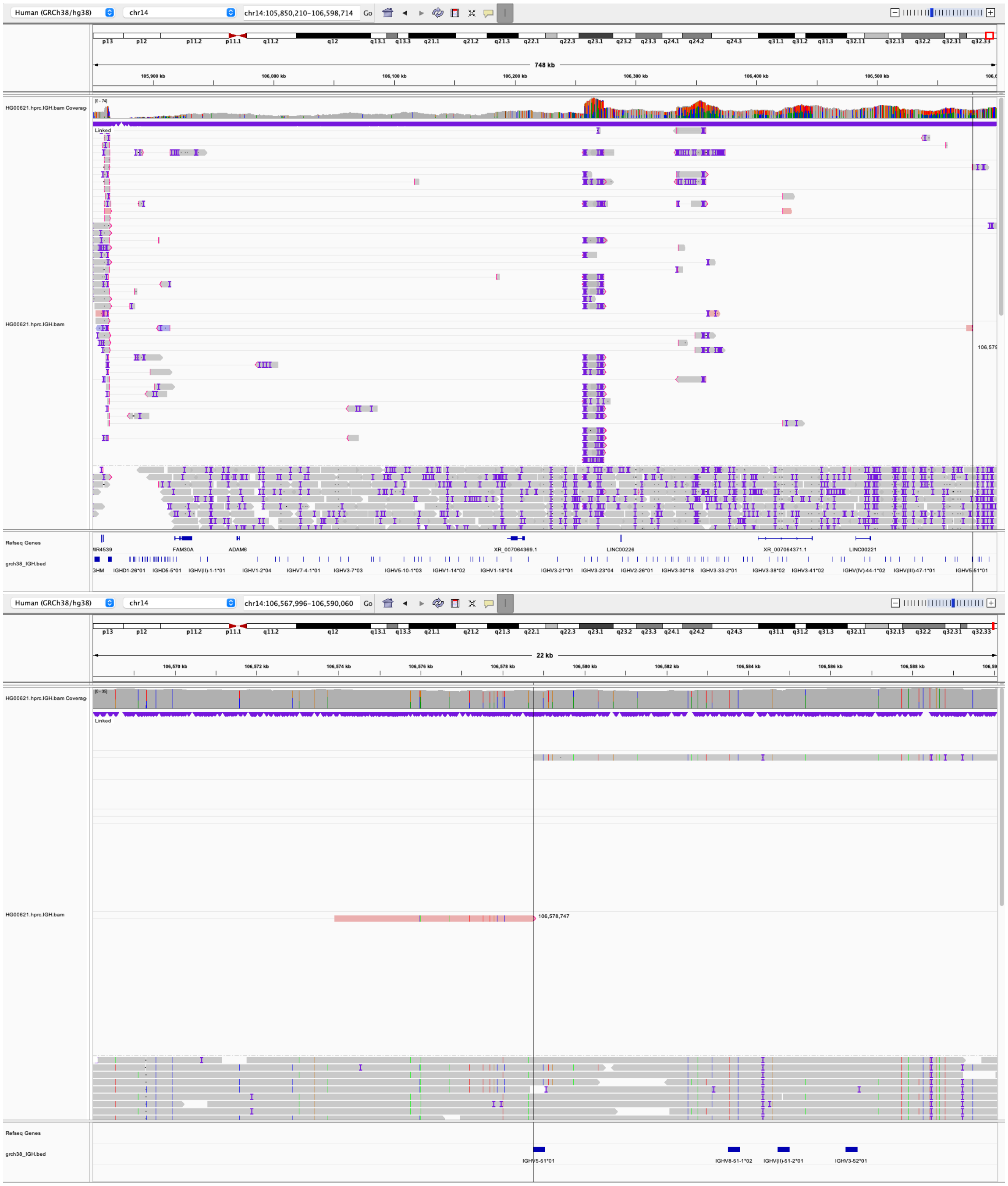

Figure S9: The bird eye view and the zoom in view of the individual HG00621 with an inverted V sequence event. The non-canonical recombination event involved an inversion starting from V gene *IGHV5-51*.

bioRxiv preprint doi: https://doi.org/10.1101/2024.07.20.604421; this version posted July 23, 2024. The copyright holder for this preprint (which was not certified by peer review) is the author/funder, who has granted bioRxiv a license to display the preprint in perpetuity. It is made available under aCC-BY 4.0 International license.

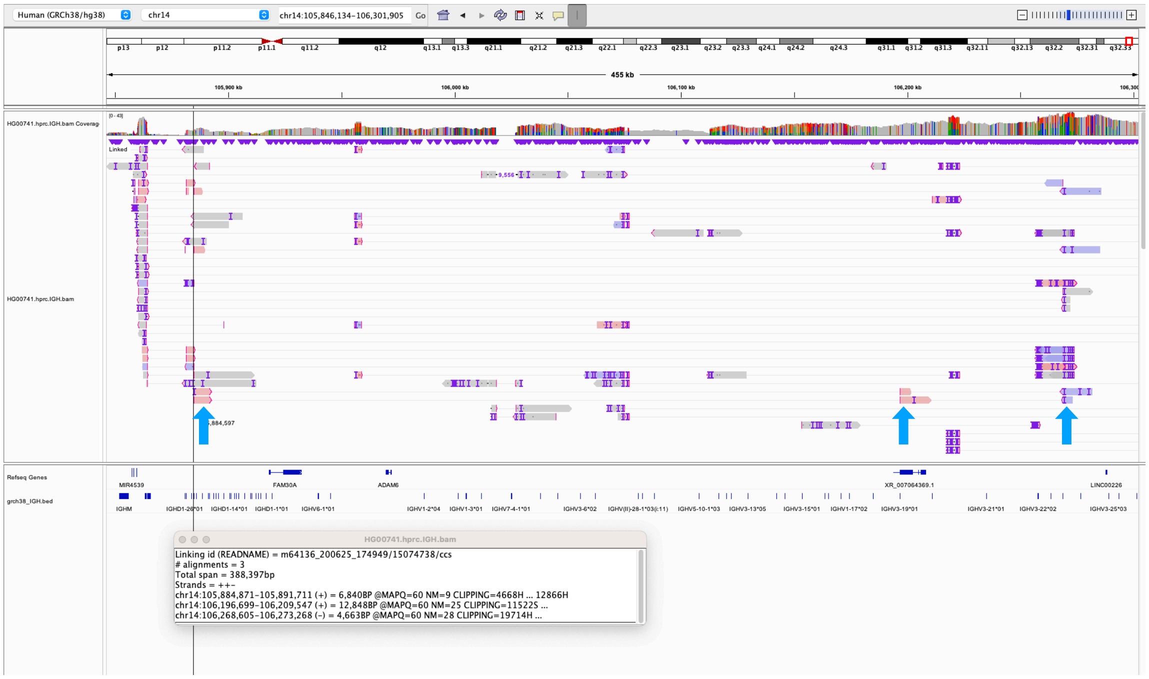

Figure S10: The IGV screenshot of the individual HG00741 with the double V-D recombination event. The segments of the non-canonical recombination event are marked with blue arrows. The event involved a V-D recombination between *IGHD6-19* and *IGHV3-19*, and another V-D recombination event between *IGHD4-23* and *IGHV3-23*.

bioRxiv preprint doi: https://doi.org/10.1101/2024.07.20.604421; this version posted July 23, 2024. The copyright holder for this preprint (which was not certified by peer review) is the author/funder, who has granted bioRxiv a license to display the preprint in perpetuity. It is made available under aCC-BY 4.0 International license.

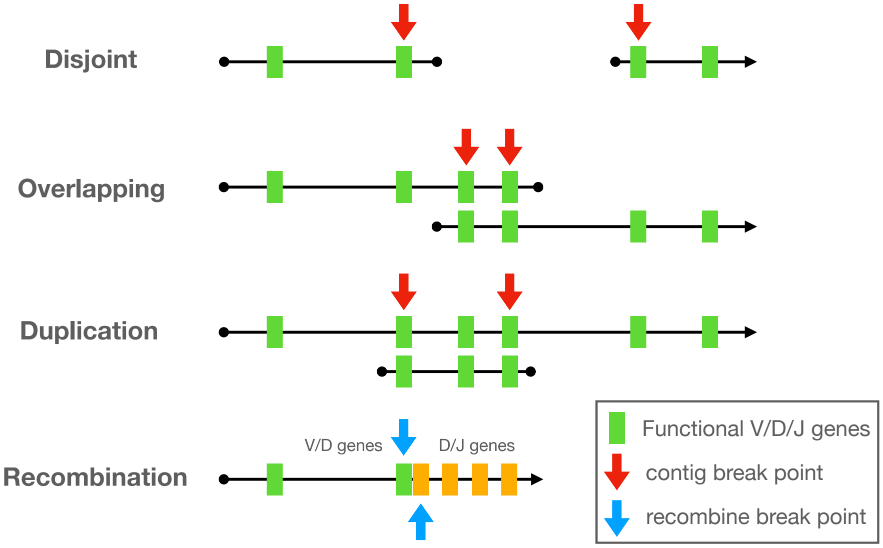

Figure S11: Categories of the breakpoints in IGH locus. **Disjoint**: the contigs end without overlapping each other. **Overlapping**: the assembler reports two separate contigs but with part of the contig overlapping each other. **Duplication**: one contig is contained in the other contig. **Recombination**: the genes changing from V to D/J, or from D to J with non-germline transition.

bioRxiv preprint doi: https://doi.org/10.1101/2024.07.20.604421; this version posted July 23, 2024. The copyright holder for this preprint (which was not certified by peer review) is the author/funder, who has granted bioRxiv a license to display the preprint in perpetuity. It is made available under aCC-BY 4.0 International license.

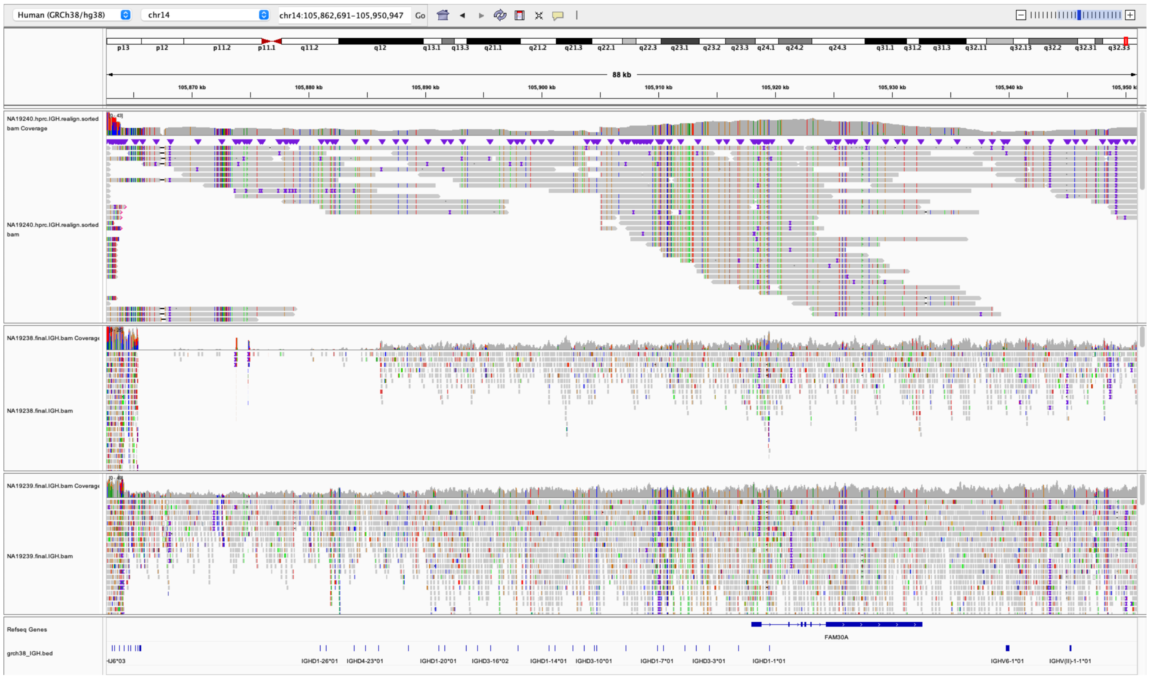

Figure S12: The IGV screenshot of the read mapping on the IGHD loci of the sample NA19240 and the sample's maternal (NA19238) and paternal (NA19239) data.

bioRxiv preprint doi: https://doi.org/10.1101/2024.07.20.604421; this version posted July 23, 2024. The copyright holder for this preprint (which was not certified by peer review) is the author/funder, who has granted bioRxiv a license to display the preprint in perpetuity. It is made available under aCC-BY 4.0 International license.

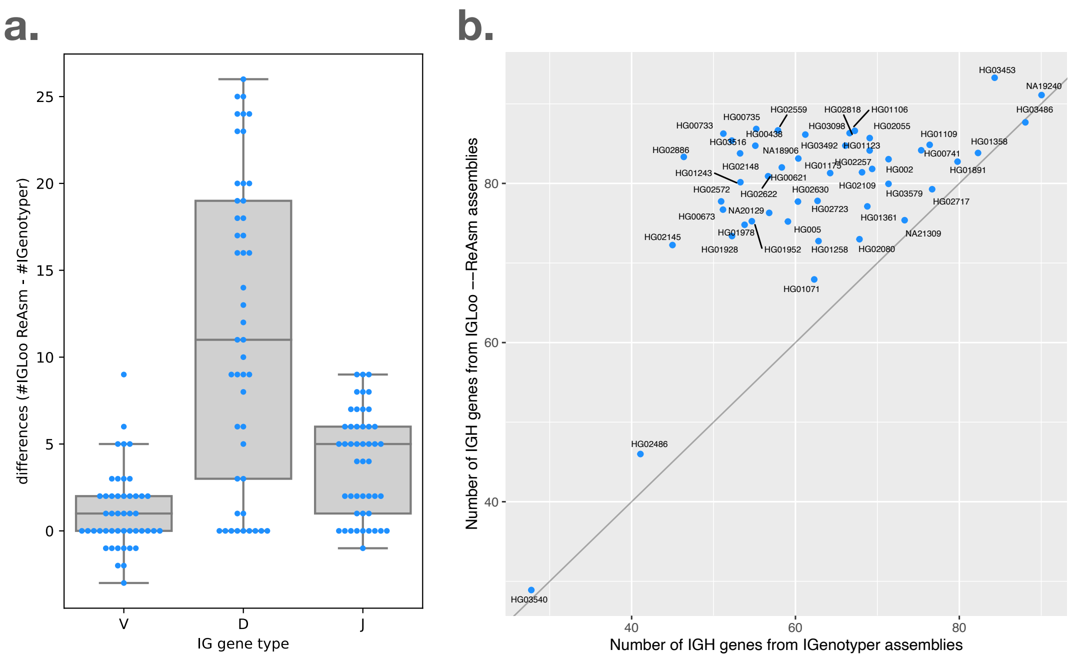

Figure S13: The IGH gene number comparison between IGenotyper (14) and `IGLoo --ReAsm` in the 47 HPRC samples. **a.** the V, D, and J gene differences between two methods in IGH locus. The positive value means `IGLoo --ReAsm` assemblies cover more IGH genes while the negative value means IGenotyper assemblies cover more genes. **b.** the total number of IGH V, D, and J genes comparison of the two methods.

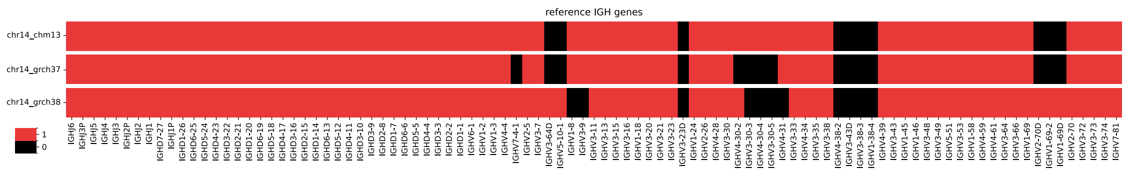

Figure S14: the gene number in its relative position on the chromosome 14 of reference genomes T2T-CHM13, GRCh37, and GRCh38. All D and J genes are listed, but only functional or ORF V genes are listed for simplicity
